# A case of carotid web: Cause of stroke in healthy and young patients

**DOI:** 10.4102/sajr.v26i1.2291

**Published:** 2022-01-28

**Authors:** Sanjay M. Khaladkar, Darshana Dilip, Rahul Arkar, Vijetha Chanabasanavar, Purnachandra Lamghare

**Affiliations:** 1 Department of Radiodiagnosis, Dr. DY Patil Medical College, Hospital and Research Centre, Pune, India

**Keywords:** carotid web, atypical fibromuscular dysplasia, cerebral angiography, recurrent cerebrovascular accident, unilateral or bilateral, embolic stroke, non-atheromatous, carotid stenosis

## Abstract

Carotid webs are important, often undiagnosed causes of cryptogenic and recurrent strokes. CT angiography and digital subtraction angiography adequately demonstrate webs as linear filling defects in the carotid bulb. However, findings are overlooked unless viewed in optimal planes and easily misdiagnosed as dissection flaps or atheromatous plaques, altering management and outcome. A case of unilateral carotid web is presented, detected during imaging in a young lady presenting with hemiparesis without other risk factors for stroke.

## Introduction

Carotid web is a term used by radiologists for describing a membranous filling defect in the carotid bulb, seen in its posterior wall. It was first used in 1973 to describe a poorly understood entity causing non-atheromatous internal carotid artery (ICA) stenosis. Since then, they have been variably referred to as carotid bulb shelf, diaphragm, septum, pseudo-valvular folds and atypical fibro-muscular dysplasia (FMD)/hyperplasia.^1^ Regardless of the nomenclature, they have been found to be an important causative factor in cryptogenic and recurrent strokes, especially in young and otherwise healthy patients.

## Case report

A 44-year-old women presented to our centre, 8 h after acute onset of left upper and lower limb weakness, associated with ipsilateral facial weakness. She had no other neurological complaints, fever, trauma, vomiting or headache. There was no previous history of transient ischaemic attacks (TIAs), angina, hypertension, dyslipidaemia, diabetes or surgeries. The patient was a teetotaller and non-smoker. Examination revealed an average build woman with a body mass index of 20.5 kg/m^2^; normal vital parameters; grade 2 power in the left upper and lower limbs with hyperreflexia, an extensor plantar reflex on the left side and deviation of the angle of mouth to the left. Clinically, these features indicated an acute stroke, likely related to a right middle cerebral artery (MCA) territory infarct.

**FIGURE 1:**
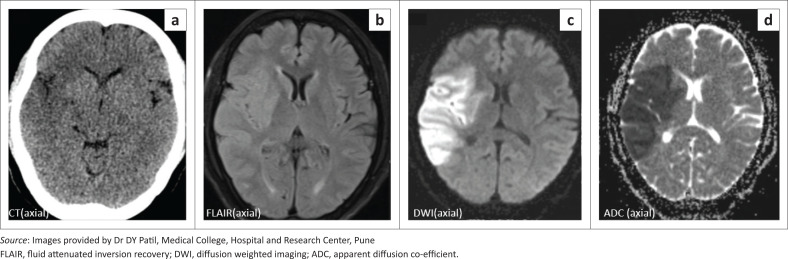
(a) NCCT brain illustrating an ill-defined hypodense infarcted area in the right fronto-parieto-temporal region, with loss of the insular ribbon and effacement of the adjacent sulci and right sylvian cistern. (b) MRI brain revealed a FLAIR hyperintense acute non-haemorrhagic infarct in the right MCA territory. DWI (c) and ADC (d) shows diffusion restriction with corresponding low ADC values.

Initial non-contrast computed tomography (NCCT) of the brain demonstrated an acute non-haemorrhagic infarct in the right parieto-temporal region, with regional loss of grey-white matter differentiation and effacement of the sulci (Figure 1a).

**FIGURE 2:**
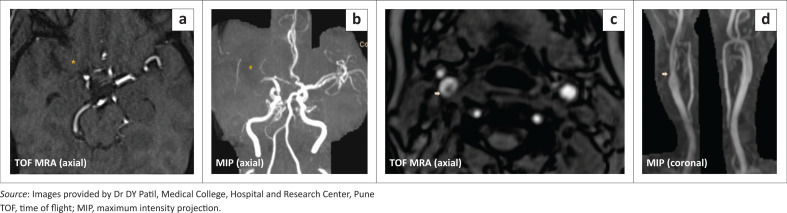
Magnetic resonance angiography TOF axial (a) and reformatted axial (b) demonstrating absent flow in all segments of the right MCA (star). Axial plane MRA of the neck vessels (c) revealed a curvilinear intra-luminal filling defect along the posterior wall of the right ICA at its origin (arrow) – suspicious of possible dissection or focal atherosclerotic plaque/thrombus. MIP coronal (d) exhibits subtle narrowing at the right carotid bifurcation.(arrow)

Magnetic resonance imaging (MRI) of the brain revealed fluid attenuated inversion recovery (FLAIR) hyperintense signal and restricted diffusion in the right MCA territory, confirming the initial diagnosis (Figure 1b, c, d). In addition, at MRA, the right MCA was not visualised along its entire length, indicating likely thrombosis (Figure 2a, b). Furthermore, a suspicious curvilinear filling defect was detected along the posterior wall of right ICA, distal to the carotid bifurcation, in a single transverse section (Figure 2c).

**FIGURE 3:**
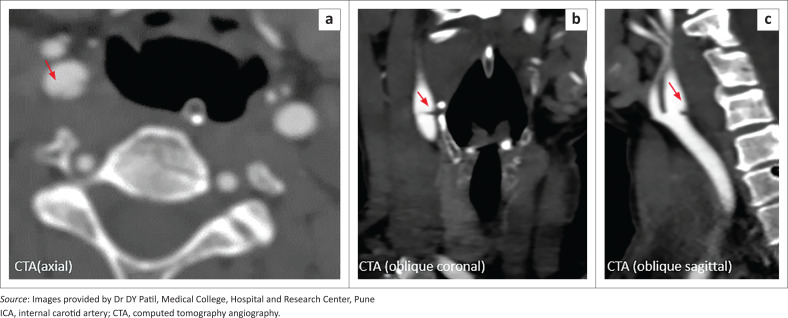
Axial (a) computed tomography angiography (CTA) demonstrating a subtle linear flap (arrow) at the posterior wall of right ICA origin. Oblique (b) and sagittal (c) reformats of the CTA indicating a distinct shelf-like 2 mm thickness filling defect, protruding into the right ICA lumen from the posterior wall, at its origin, suggestive of a carotid web.

Computed tomography angiography (CTA) was acquired to accurately characterise the filling defect observed in the right ICA and it successfully depicted a partial circumferential shelf-like filling defect, measuring 2 mm × 8 mm, along the posterior wall of right ICA at its origin – establishing the diagnosis of a carotid web (Figure 3a and b). Thrombosis of the right MCA was confirmed. The left ICA and the rest of the major head and neck vessels were normal.

**FIGURE 4:**
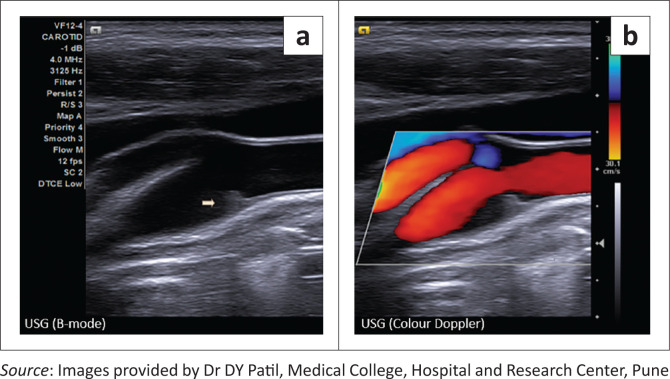
(a) Ultrasound of right carotid bulb in longitudinal section revealed a shelf-like intimal projection from the posterior wall (arrow); no calcification or thrombus seen. (b) Patent flow on colour Doppler.

Doppler screening of the neck vessels revealed a focal intimal projection at the same site, not causing significant alteration to the flow of blood in the vessel (Figure 4a and b). There was no evidence of a lodged thrombus at the carotid web.

The patient was unwilling to undergo invasive imaging or definitive management by stenting or endarterectomy at our interventional radiology department. Currently, she is being treated conservatively with dual oral antiplatelet therapy, anticoagulant injections and statins.

## Discussion

Carotid webs are non-atheromatous bands of 1 mm – 2 mm thickness seen along the posterior wall of proximal ICA. The reported incidence of carotid web is less than 1%, which is likely an underestimation, as the majority of diagnosed carotid webs have been in patients imaged for stroke. Carotid bulb webs more commonly affect women.^2^ While the precise aetiology is uncertain, genetic and hormonal influence, oral contraceptive use, chronic intimal insult and abnormal trophoblastic activity are all likely factors in the development of carotid webs.^3^ Histologically, they are formed by fibrosis, myxoid degeneration and smooth muscle hyperplasia of the tunica intima. Hence, they have also been called atypical FMD, in contrast to typical FMD, which affects the tunica media of the renal, vertebral, mid to distal carotid arteries.^1,4^

Stasis of blood downstream to the carotid web results in a thrombogenic niche, which leads to recurrent systemic embolism, presenting as TIA or stroke, most commonly affecting the ipsilateral MCA or anterior cerebral artery (ACA) territories. Causation is not identified in roughly a third of all cases of stroke, referred to as cryptogenic strokes. Sajedi et al. reported that 21.2% cases of cryptogenic stroke had carotid webs, with a fairly uniform distribution of cases with unilateral and bilateral pathology in the study group. In addition, a lesser mean age for stroke was detected in patients with a carotid web (38.3 years) than among those without webs (48.7 years).^4^ Hu et al. determined a strong association between carotid webs and TIA in patients having no other vascular risk factors. They also reported a staggering 83.3% short-term recurrence rate of stroke/TIA among patients having carotid webs versus patients without carotid webs (12.2%).^5^ Thus, patients with carotid webs suffering cardiovascular accidents were younger, commonly female and less frequently had vascular risk factors such as hypertension, diabetes, dyslipidaemia or smoking.^6^

Digital subtraction angiography (DSA) because of its higher spatial and temporal resolution, has till date been the accepted gold standard in diagnosing carotid webs. However, recent studies have proven equivalent performance by CTA with multiplanar reformatting. This is over and above the general advantages of CTA being cost-effective, easily available and non-invasive. Magnetic resonance angiography, although superior in delineating vessel wall anatomy, has lower sensitivity and specificity compared with CTA and DSA.^3,4,6^

At ultrasound, carotid webs appear as echogenic intimal projections from the posterior carotid bulb, which are less reliably distinguished from atherosclerotic plaques.^6^ However, the degree of stenosis is more accurately determined by ultrasonography and haemodynamic alterations are better assessed using Doppler.

Carotid webs appear as triangular, linear or membranous filling defects protruding into the lumen from the posterior wall of the proximal ICA or carotid bulb at CTA, DSA and MRA. Contrast pooling maybe observed within the web at DSA. Calcification or thickening should be absent within 3 mm of its location. Associated thrombosis may be seen as a focal filling defect lodged downstream of the web. As a result of their film-like thinness (1 mm – 2 mm) and transverse placement, they are seen as a subtle septum on axial views. Therefore, oblique sagittal views obtained by CTA multiplanar reformats are the best tool for identification.^1,2,3,4^

Carotid dissection, focal atherosclerotic plaques (FAP) and typical FMD are the imaging mimics of carotid webs.^2,3^ Focal atherosclerotic plaque appears as an irregular concentric calcification or hypoattenuating wall thickening, causing variable degrees of luminal stenosis and proportional velocity changes on Doppler, findings, which are not seen with a carotid web.^2^ Dissection flaps typically propagate distally from the carotid bulb, have irregular borders, may show intra-mural haematoma or vessel widening related to pseudoaneurysm formation and have the classic ‘double lumen’ appearance.^3,4,7^ Typical FMD is usually multisegmental, giving the characteristic string of beads appearance to the vessel.^1,2,3^

Opinions on optimal management of carotid webs vary because of lack of clear guidelines at present. Prophylactic dual antiplatelet and anticoagulant therapies are being used in the setting of an acute stroke/TIA and in some asymptomatic cases. Nevertheless, they have been found to be ineffective in preventing recurrent cerebrovascular accidents in about 30% cases.^3,7,8^ Hence, carotid stenting or endarterectomy have been favoured by physicians for treating carotid webs in symptomatic cases, with no consensus as to which is the optimal curative option.^7,8^

## Conclusion

Carotid webs are a cause of recurrent stroke, especially among young patients with no other vascular risk factors. A high clinical suspicion should be borne in mind during imaging cases of cryptogenic stroke. To aid in accurate diagnosis, multimodality imaging using CTA or DSA should be performed for suspicious lesions that have been detected at ultrasound or MRA. Timely diagnosis can have long-reaching effects in disability prevention and survival among patients.
